# *Staphylococcus simulans*: A rare uropathogen

**DOI:** 10.1016/j.idcr.2021.e01202

**Published:** 2021-06-17

**Authors:** Ana Drobeniuc, Jessica Traenkner, Paulina A. Rebolledo, Varduhi Ghazaryan, Nadine Rouphael

**Affiliations:** a Hope Clinic of the Emory Vaccine Center, Division of Infectious Diseases, Department of Medicine, School of Medicine, Decatur, GA, USA; b Division of Microbiology and Infectious Diseases, National Institute of Allergy and Infectious Diseases, National Institutes of Health, Bethesda, MD, USA

**Keywords:** Complicated urinary tract infections, *Staphylococcus simulans*, Zoonoses

## Abstract

Urinary tract infections (UTIs) are clinically and economically burdensome. Gram positive causative uropathogens are rare, and *Staphylococcus simulans* has infrequently been isolated as a causative agent for UTIs. Here, we present two cases of *S. simulans* causing complicated urinary tract infections.

## Introduction

UTIs contribute yearly to a high number of urgent care center, emergency department, and hospital admissions leading to a high economic burden [1,2]. Frequently in an outpatient setting, the diagnosis is based on clinical presentation and urinalysis; therefore, empirical therapy is prescribed either without a urine culture and susceptibility testing or before such results are known [3].

UTIs are generally due to gram-negative bacteria. Among the most common gram positive uropathogens are coagulase-negative staphylococci (CoNS) and *Enterococcus*. Rarely do other gram positive pathogens cause UTIs in the absence of urinary catheters or disseminated infections. CoNS more broadly have been identified as causative uropathogens in 3% of outpatient and 13 % of inpatient cases, most notably *S. saprophyticus* [4] which is part of the commensal flora of the female genital tract. In contrast *S. simulans,* a common animal pathogen, is rarely found on human skin and has only been isolated in 0.2%–2.1% of urinary specimens [[5], [6], [7], [8]].Traditionally, the identification process for gram positive cocci is based on microscopic and colony morphology as well as a catalase test. Although both *S. saprophyticus* and *S. simulans* are identified as CoNS using the Gram stain and catalase test, identification to the species level rely on automated systems or specific biochemical testing, often delaying the diagnosis by several days. In recent years, matrix assisted laser desorption ionization time of flight spectrometry (MALDI-TOF MS) has become an important tool not only for rapid microbial identification but also for the ability to accurately differentiate between species of CoNS [9]. Evolving practices for microbial identification in the laboratory using newer technologies could potentially result in more frequent identification of *S. simulans* as a cause of human disease.

To the best of our knowledge, no case reports of complicated UTIs (cUTIs) caused by *S. simulans* have been published. Herein, we present two cases of cUTIs caused by *S. simulans*.

## Case 1

61 year-old female with history of recurrent UTI and kidney stones presented to the urgent care center with complaints of bilateral flank pain, hematuria, dysuria, nausea, subjective fever, and chills for three weeks. Social history was significant for living on a farm in Georgia. On physical exam she was afebrile and had bilateral CVA tenderness. The patient had mild leukocytosis of 12,600/mcL. Urinalysis revealed positive nitrites, 3+ leukocyte esterase, and >50 white blood cells with a urine culture with greater than 100,000 CFU/mL of *S. simulans*. CT scan of the abdomen and pelvis revealed a 5 mm non-obstructing calculus of the left renal pelvis and an 8 mm obstructing calculus at the left ureteropelvic junction with mild hydroureteronephrosis and trace stranding. She was given ceftriaxone followed by sulfamethoxazole-trimethoprim to which the uropathogen was susceptible. The pathogen was resistant to fluoroquinolones. She underwent cystoscopy with left ureteral stent placement. Patient returned 10 days post-discharge for left ureteroscopy, laser lithotripsy, and stent exchange with resolution of her cUTI symptoms. No further follow up was observed per the medical record.

## Case 2

56 year-old male with a history of paraplegia secondary to a gunshot wound in 1997 presented to the emergency department with complaints of dysuria, malodorous urine, urinary frequency, abdominal pain, and nausea. His medical history was significant for urinary retention and prior UTIs due to gram negative organisms. Patient reported visiting family farm several months prior to hospitalization. His physical exam was significant for bilateral CVA tenderness and suprapubic tenderness. Urinalysis revealed 3+ leukocyte esterase, and >50 white blood cells. His urine culture resulted in greater than 100,000 CFU/mL *S. simulans* resistant to fluoroquinolones. Patient was treated with ceftriaxone, then was given fosfomycin to which he developed diarrhea and then completed a 10-day course of oral sulfamethoxazole-trimethoprim to which the pathogen was susceptible. He was seen in the ED one month later for residual lower extremity edema; however, patient did not report urinary symptoms.

## Discussion

*Staphylococcus simulans*, a coagulase-negative staphylococcus (CoNS), is rarely found to colonize human skin. It is an opportunistic pathogen among animals (cows, goats, horses, and other farm or domesticated animals) and has been frequently associated with bovine mastitis [10]. Spread of coagulase negative staphylococci has been described in animals and personnel at equine facilities [11]. Therefore, a detailed social history of exposure to farm animals should raise the suspicion of *Staphylococcus simulans* as a potential uropathogen.

Though rare, human infections caused by *S. simulans* have been reported, and include soft tissue infections, endocarditis, osteomyelitis, bacteremia, and includes singular reports of pleural empyema, pneumonia, and corneal infections [[12], [13], [14], [15], [16], [17], [18]]. *S. simulans* has rarely been isolated from urinary specimens of patients [5,7]. There were no detailed case reports of UTIs caused by *S. simulans*, however, Razonable et al. describe a case of vertebral osteomyelitis and prosthetic joint infection, which was immediately preceded by a UTI, speculated as a potential source of infection. However, urine culture results in this case were unavailable [14]. In our cases, the microbiology lab confirmed the result as a pure culture with no co-pathogens isolated by MALDI-TOF system.

For cUTI, results of urine culture and susceptibility testing should be used to confirm that the chosen empiric regimen is active or to tailor the regimen, if appropriate. CoNS more broadly have been identified as causative cUTI pathogens in less than 3% of cases, most notably *S. saprophyticus* [19]. Quinolones are standard of care for cUTI; however, they have variable antimicrobial activity against staphylococci isolates. Sulfamethoxazole-trimethoprim has good coverage of both gram-negative and gram-positive uropathogens but is typically used for the empiric treatment of uncomplicated UTIs and not for cUTIs [20].

A detailed social history is indicated in patients with UTIs. Evolving practices for microbial identification in the laboratory using newer technologies could potentially result in more frequent identification of *S. simulans* as a cause of human disease.

## Funding

NIH/NIAID/DMID awards to the Emory Vaccine and Treatment Evaluation Units [VTEU]: HHSN272201300018I, HHSN27200003, and HHSN27200018. NIH/NIAID/DMID did not participate in the collection, analysis, or interpretation of data, in the writing of the manuscript or in the decision to submit the manuscript for publication.

## Ethical approval

Not applicable.

## Disclaimer

The opinions expressed in this article are those of the authors and do not reflect the view of the National Institute of Allergy and Infectious Diseases, the National Institutes of Health, the Department of Health and Human Services, or the United States Government.

## Consent

Written informed consent was obtained from the patients for publication of this case report. Copies of both written consents are available for review by the Editor-in-Chief of this journal on request.

## Author contribution

**Ana Drobeniuc:** Data collection, writing.

**Jessica Traenkner:** Conceptualization, data collection, writing.

**Paulina A. Rebolledo:** Conceptualization, data collection, writing.

**Varduhi Ghazaryan:** Conceptualization.

**Nadine Rouphael:** Conceptualization, writing, supervision, funding acquisition.

## Declaration of Competing Interest

The authors report no declarations of interest.
